# Erythropoietin-mediated activation of aquaporin-4 channel for the treatment of experimental hydrocephalus

**DOI:** 10.1007/s00381-018-3865-z

**Published:** 2018-07-08

**Authors:** M. Rizwan Siddiqui, Furqan Attar, Vineet Mohanty, Kwang Sik Kim, C. Shekhar Mayanil, Tadanori Tomita

**Affiliations:** 1 Pediatric Neurosurgery Research Program, Stanley Manne Children’s Research Institute, Ann & Robert H. Lurie Childrens’ Hospital of Chicago, Northwestern University Feinberg School of Medicine, Chicago, IL USA; 2 Division of Pediatric Infectious Diseases, Johns Hopkins University School of Medicine, Baltimore, MD USA; 3 Division of Pediatric Neurosurgery, Ann & Robert H. Lurie Children’s Hospital of Chicago, Northwestern University Feinberg School of Medicine, Chicago, IL USA

**Keywords:** Hydrocephalus, Erythropoietin, Aquaporin water channel, MicroRNA

## Abstract

**Objective:**

In this study, we investigate a neuroprotective agent, erythropoietin (EPO), in animal hydrocephalus model and its potential reversal effects on hydrocephalus by altering the expression of aquaporin-4 (AQP4).

**Methods:**

Obstructive hydrocephalus was induced in 2-week-old rat pups by injecting kaolin (50 μl, 10 mg/ml in saline) into the cisterna magna, while the control pups received only saline. Kaolin-injected pups were divided into two groups on the fifth day after kaolin injection; one group received intra-peritoneal (i.p.) EPO (1 μg/pup) for 5 consecutive days, while other group received i.p. saline for 5 days. The effects of EPO on hydrocephalus were investigated by studying cerebral ventricle size and structural ependymal changes. We examined also the EPO effects on AQP4 expression and microRNA expression.

**Results:**

EPO treatment significantly reduced dilation of the cerebral ventricle and denudation of ependymal line in hydrocephalic pups comparing with the control group. Increased expression of AQP4 in periventricular ependymal lining and cultured astrocytes and increased vascular formation were noted after EPO treatment. Additionally, we identified miR-668 as an endogenous regulator of AQP4 in response to EPO. Anti-miR-668 dampened EPO-induced activation of AQP4 expression.

**Conclusions:**

Together, our results show that EPO-mediated upregulation of AQP4 significantly reduces dilation of the cerebral ventricles in obstructive hydrocephalus pups and may lead to potential therapeutic options for hydrocephalus.

## Introduction

Current established treatment of hydrocephalus has been surgical, including elimination of its cause or CSF diversion. However, despite technical advancement, failures of CSF diversion are common and approximately 40% children treated with a shunt need to have some intervention or shunt revision within 2 years of the original shunt insertion [1, 2]. Non-surgical therapies for hydrocephalus have been proposed and practiced so far with limited success [3]. Therefore, advanced and alternative non-surgical treatments for hydrocephalus are needed.

Aquaporin (AQP) is a water-transporting protein present in plasma membrane and transports water in and out of the cell. AQP1, AQP4, and AQP9 are expressed in brain where they are involved in water homeostasis. AQP4 is the principal, most abundant water channel in the brain providing a low-resistance transcellular clearance route and regulating water flow in and out of the brain. It is concentrated on astrocytic end-feet processes at CSF–brain interfaces (Virchow Robin’s space, glia limitans interna, and glia limitans externa) and at blood–brain interfaces [4–8]. Hydrocephalus upregulates brain water channel AQP4 at the brain–CSF interfaces as well as surrounding astrocyte end-feet and subpial layers in hydrocephalus animals [9]. The upregulation of AQP4 expression facilitates increased water passage through the brain back into the vasculature [4]. Thus, AQP4 channels play a role in trans-parenchymal water clearance in obstructive hydrocephalus from the ventricle and extracellular space of the brain into the cerebral vasculature [10, 11].

AQP4 channels are mainly located on astrocyte end-feet but is later distributed over the whole membrane of astrocytes when hydrocephalus progresses [12], suggesting that upregulated AQP4 expression is a physiological adaptation to induced hydrocephalus, possibly to aid and facilitate removal of excess water. Water effluxes via glial-vascular pathway or “glymphatic pathway” [13] by modulating AQP4 channel is an attractive therapeutic target for potential molecular treatment of hydrocephalus.

Erythropoietin (EPO) is an endogenous hematopoietic cytokine, which has anti-inflammatory and angiogenetic effects. EPO binds the EPO receptor, which is widely expressed in the brain [14]. One of the mechanisms through which EPO reduces brain edema is by regulating expression of the AQP4 water channel. EPO significantly upregulates AQP4 expression in brain tissue and reduces injury in hypoxia [15] and leads to a better clearance of water excess in brain tissue mediated by AQP4 [16]. Moreover, EPO can also induce angiogenesis while inhibiting vascular leakage and inflammation [17].

The role of microRNAs in the expression of AQP4 has recently been studied. Interaction of hsa-miR-668, -1280, -130a, -152, and -939 was noted with the promoter of the AQP4 M1 gene [18. 19]. Among them, miR-130a appears to be a strong suppressor of promoter activity. Previous studies showed that erythropoietin (EPO) delivers protective effects by regulating microRNA (miRNA) expression through their interaction [20, 21]. Because miR-130a functions as a suppressor that can modulate promoter activity, anti-miR-130a could serve as a potential therapeutic agent in ischemic recovery [22].

We therefore hypothesize that the severity and duration of hydrocephalus can be rescued by EPO-mediated upregulation of AQP4 by modifying CSF flow into the cerebral blood stream through AQP4 channels. Given the importance of the role of AQP4 in brain edema, we also explored the possibility of identifying miRNAs downstream of EPO signaling that could selectively regulate AQP4 expression.

## Material and methods

### Animal procedure

Sprague Dawley rats were purchased from Charles River. Female pups of 2 weeks in age with body weight 20–22 g were used in this study. A total of 32 pups were used in this study. Pups were anesthetized by giving isoflurane inhalation. Hydrocephalus was induced in 22 pups through injection of kaolin suspension (50 μl, 10 mg/ml in sterile saline) with 30-gauge syringe into cisterna magna. In control group, pups were injected with 50 μl of saline in cisterna magnum. Kaolin-injected animals were equally divided in to two groups. At the fifth day of kaolin cisternal injection, one group of pups was injected with mouse recombinant EPO (R&D Systems, Minneapolis, MN) 1 μg/pup, intra-peritoneal (i.p.) for 5 consecutive days, while the other was injected with saline for 5 days. All pups were observed throughout recovery from anesthesia and housed in a controlled environment with ad libitum access to food and water. Their behavior and body weight were recorded on daily basis as per Olopade et al. [23]. At 10th day of post kaolin injection, all pups were sacrificed with institutional guidelines and regulations.

### Cell culture

Human astrocytes were obtained from Dr. Rintaro Hashizume, Northwestern University Chicago, IL, USA. Rat choroid plexus epithelial cell line Z310 was provided by Dr. Wei Zheng, Purdue University, West Lafayette, IN, USA. Z310 cells were cultured in RPMI 1640 medium, and the astrocyte cell line was cultured in Eagle’s modified Eagle’s medium. The medium was supplemented with 10% fetal bovine serum, 50 units of penicillin, and 50 μg/ml streptomycin. Human brain microvascular endothelial (HBME) cells were cultured as described previously [20]. All cells were cultured in T-75 flasks and maintained in a 37 °C incubator with 5% CO_2_.

### Anti-miRNA transfection

Transfection with anti-miR-668 and scrambled anti-miR miRNA Inhibitor Negative Control #1 from Applied Biosystems was performed using Lipofectamine RNAiMAX transfection reagent (Life Technologies). Cells were seeded to 80% confluency prior to transfection. For the transfection, anti-miRNA-lipid complexes were formed using Opti-MEM medium, Lipofectamine RNAiMAX transfection reagent, and respective miRNA inhibitor. Per well, the final concentration of miRNA inhibitors added amounted to 50 nmol for cells on a six-well plate. After 48 h of transfection, cells were left untreated or treated with EPO for different time points. Total RNAs including miRNA were isolated to examine mRNA expression of AQP-4 and for miRNA quantification. In other experiment, cells were lysed in RIPA buffer for immunoblot analysis of AQP-4 protein expression.

### Reverse transcription and quantitative real-time PCR

Reverse transcription followed by real-time quantitative PCR (qRT-PCR) was carried out according to Mohanty et al. [24]. Quantitation of AQP4 mRNAs was performed using a SYBR Green assay with forward primer, 5-agcctgggatgcaccatca-3, and reverse primer, 5-tgcaatgctgagtccaaagc-3. miRNA from cells was isolated by using the miRNAeasy Mini Kit (Qiagen). Subsequently, cDNA generation was accomplished using the TaqMan MicroRNA Reverse Transcription Kit (Applied Biosystems, Foster City, CA). Following assembly of all components for cDNA synthesis, the reverse transcription reaction was carried out in a thermocycler. TaqMan Universal Master Mix II, no UNG (Applied Biosystems) was used in preparing reactions for qPCR. TaqMan microRNA assay kits from Applied Biosystems were used for miRNA quantified by q-RT-PCR. Data collection for each cycle was done at the completion of the annealing/ extension step. RT primers (for cDNA synthesis) and TM primers (for qPCR) for U6 snRNA control, hsa-miR-130a, hsa-miR-let-7f, hsa-miR-668, and hsa-miR-320a were obtained from Applied Biosystem’s TaqMan. All the real-time PCR experiments were carried out using an Applied Biosystems 7900 sequence detection system.

### Western blot analysis

Western blotting using 30 μg of total proteins was carried out as described by Siddiqui et al. [20]. Membrane was probed with rabbit anti-AQP4 antibody (Abcam) at a concentration of 1 μg/ml in 4% blocking solution. Mouse anti-actin antibody (Santa Cruz Biotechnology) was used as a loading control. The membranes were visualized via enhanced chemiluminescence (Super Signal West, Thermo Scientific).

### H&E staining and immunohistochemistry

Pups were perfused with 4% paraformaldehyde and brains were collected and fixed in 10% formaldehyde. After fixation, the brains were embedded in paraffin blocks and sections were prepared (10 μm). Paraffin sections were rehydrated and stained with hematoxylin and eosin dye. For immunohistochemistry, AQP4 protein was probed with mouse anti-AQP4 antibody (dilution 1:200) and with FITC-coupled donkey anti-mouse secondary antibody (Jackson ImmunoResearch, West Grove, PA) (dilution 1:200). DAPI was used as a nuclear stain. Images were viewed at × 10 and × 63 magnifications and analyzed using LSM870 confocal imaging software (Carl Zeiss, Thornwood, NY).

### Effect of EPO on AQP4 targeting miRNAs

AQP4 targeting miRNAs were selected with Bioinformatics search in microRNA.org and Targetscan. We selected miR-130a, miR-320a, miR-let-7f, and miR-668 on the basis of previously published results [19, 20] to test whether AQP4 is regulated by these miRNAs in response to EPO. Ependymal epithelial cells derived from choroid plexus (Z310) and astrocytes were treated with EPO (20 IU/ml) for different time points. Subsequently, RNA was isolated and miR-130a, miR-320a, miR-let-7f, and miR-668 levels were determined by qPCR.

### In vitro endothelial tube formation

Cultured cerebrovascular endothelial cells (ECs) were studied for the effect of EPO on AQP4 expression and angiogenetic effects of EPO on ECs. HBME cells (5 × 10^4^) were suspended in 500 μl of serum-free DMEM media with or without EPO 20 IU/ml on a 24-well plate pre-coated with reduced factor Matrigel (Corning, NY). After 6 h of incubation at 37 °C and 5% CO_2_, pictures of the vascular tube-like structures were taken under × 20 magnifications. The number of polygonal areas formed by these tube-like structures was counted for each field.

### Statistical analysis

Comparison between different pair was done using Student’s *t* test. Comparison of three or more groups was performed using one-way analysis of variance (ANOVA). A *P* value of 0.05 or less was considered to be statistically significant.

## Results

### Effect of EPO on body weight and ventricle enlargement after kaolin-induced hydrocephalus

**Fig. 1:**
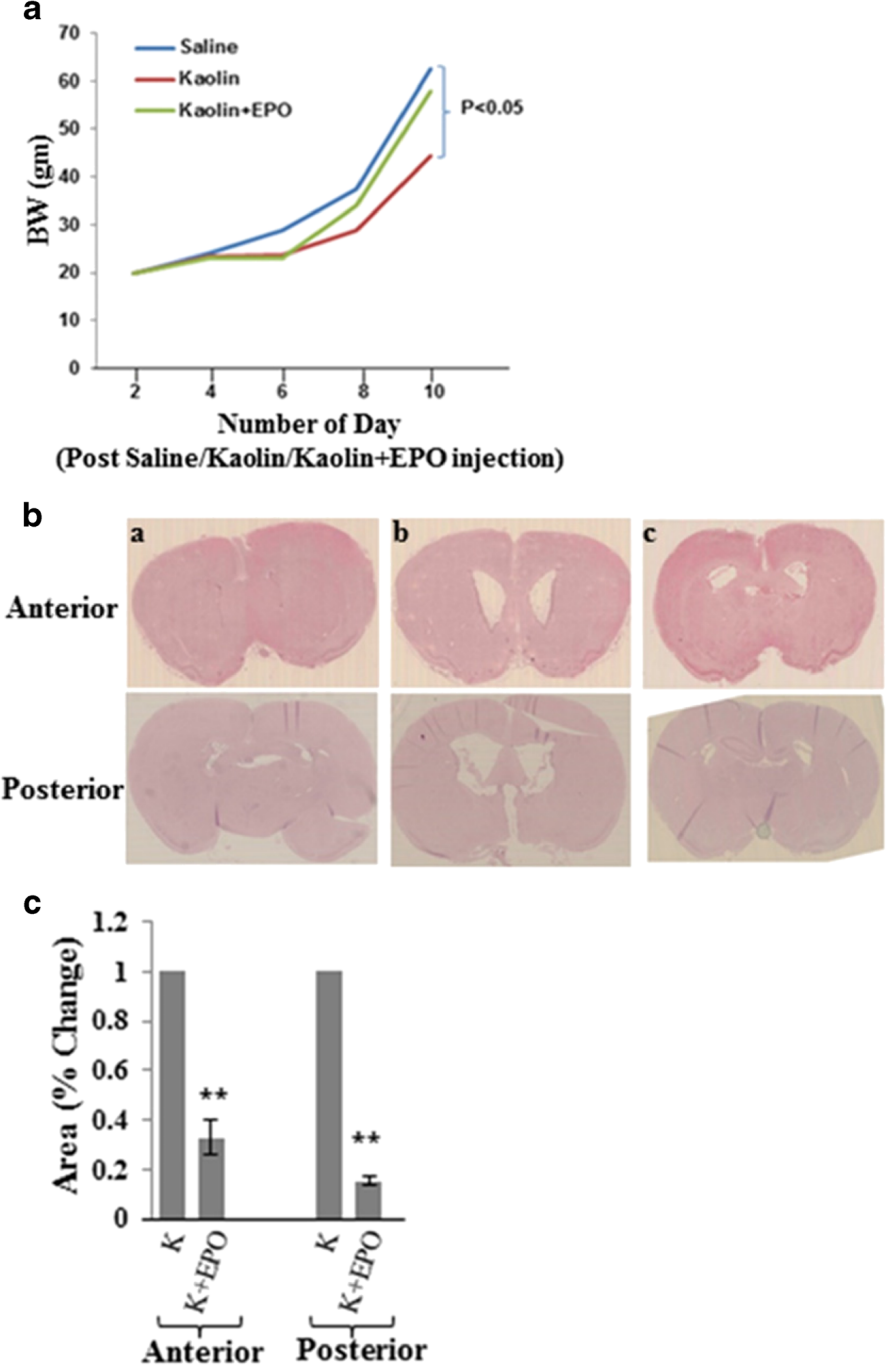
Effect of EPO treatment on body weight and ventricle enlargement following kaolin-induced obstructive hydrocephalus. (A) Body weight was determined at indicated time points in each group. Each value is the mean ± SEM of three separate experiments. *P* values are shown as *p* < 0.05 vs. saline group. (B) H&E stained brain coronal section of saline (a), kaolin (b), and kaolin-treated with EPO (c). (C) Morphometric analysis of the total area in the anterior and posterior regions of the lateral ventricle shows significant reduction in enlarged ventricular space after EPO treatment of kaolin-injected pups. **p < 0.01 vs kaolin group

The changes in body weight of pups observed in control and experimental groups are summarized in Fig. 1(A). No loss in body weight was observed in control pups whereas body weight gain was significantly reduced in pups with obstructive hydrocephalus group due to lethargy and decreased oral intakes (*p* < 0.01). EPO treatment led to significant recovery of body weight of pups as compared to saline treated pups following induction of obstructive hydrocephalus. We did not observe incidence of mortality in any animal for either the control or experimental group.

To evaluate the effect of kaolin and EPO on morphological changes and ventricular dilation, H&E staining was performed on coronal section of whole brain (Fig. 1(B)). Comparing with control group receiving saline injection alone (Fig. 1(a)), substantial enlargement of lateral ventricle in both anterior and posterior regions in the kaolin-induced hydrocephalic brains was observed (Fig. 1(b)). On the other hand, treatment of EPO significantly reduced dilation of ventricles in hydrocephalic brains following EPO i.p. treatment (Fig. 1(c)). Quantitative measurement of the ventricular space showed that the hydrocephalic pups have substantially enlarged ventricles (in both anterior and posterior regions of lateral ventricles) while EPO treatment significantly reduced kaolin-induced ventricle enlargement (Fig. 1(C)).

### Effect of EPO on ependymal defects and AQP4 expression after kaolin-induced hydrocephalus

**Fig. 2:**
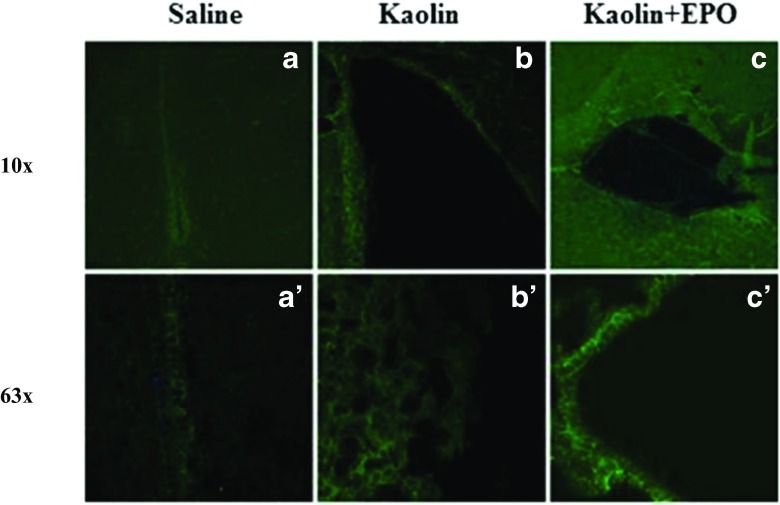
EPO treatment inhibits denudation of ependymal line and enhanced AQP4 expression. (A) Immunostaining of brain coronal section show AQP4 stained ependymal cells lined the ventricle in control (a, a’), structural anomalies were observed in kaolin-treated group (b, b’), and EPO treatment upregulates AQP4 expression and reorganizes ependyma along the ventricle (c, c’)

When compared with control group (Fig. 2(a, a’)), severe denudation of ependymal lining at the ventricular wall accompanied by clustering of ependymal cells among kaolin-induced hydrocephalus group was observed under high magnification (Fig. 2(b, b’)). In contrast, EPO treatment group showed well-organized ependymal layer (Fig. 2(c, c’)). In order to identify a critical role of AQP4 in progression of hydrocephalus, we tried to gain insights into the potential mechanisms of protective effect of EPO. By immunohistochemistry, we examined the AQP4 expression on ventricular ependymal cells in brain treated with saline, kaolin, and kaolin + EPO, respectively. No significant difference was observed in AQP4 expression between control and kaolin-induced hydrocephalus at ependymal region (Fig. 2(a’, b’)). However, we observed high expression of AQP4 after EPO treatment in kaolin-injected brain (Fig. 2(c’)).

### AQP4 expression in cultured astrocytes to EPO

**Fig. 3:**
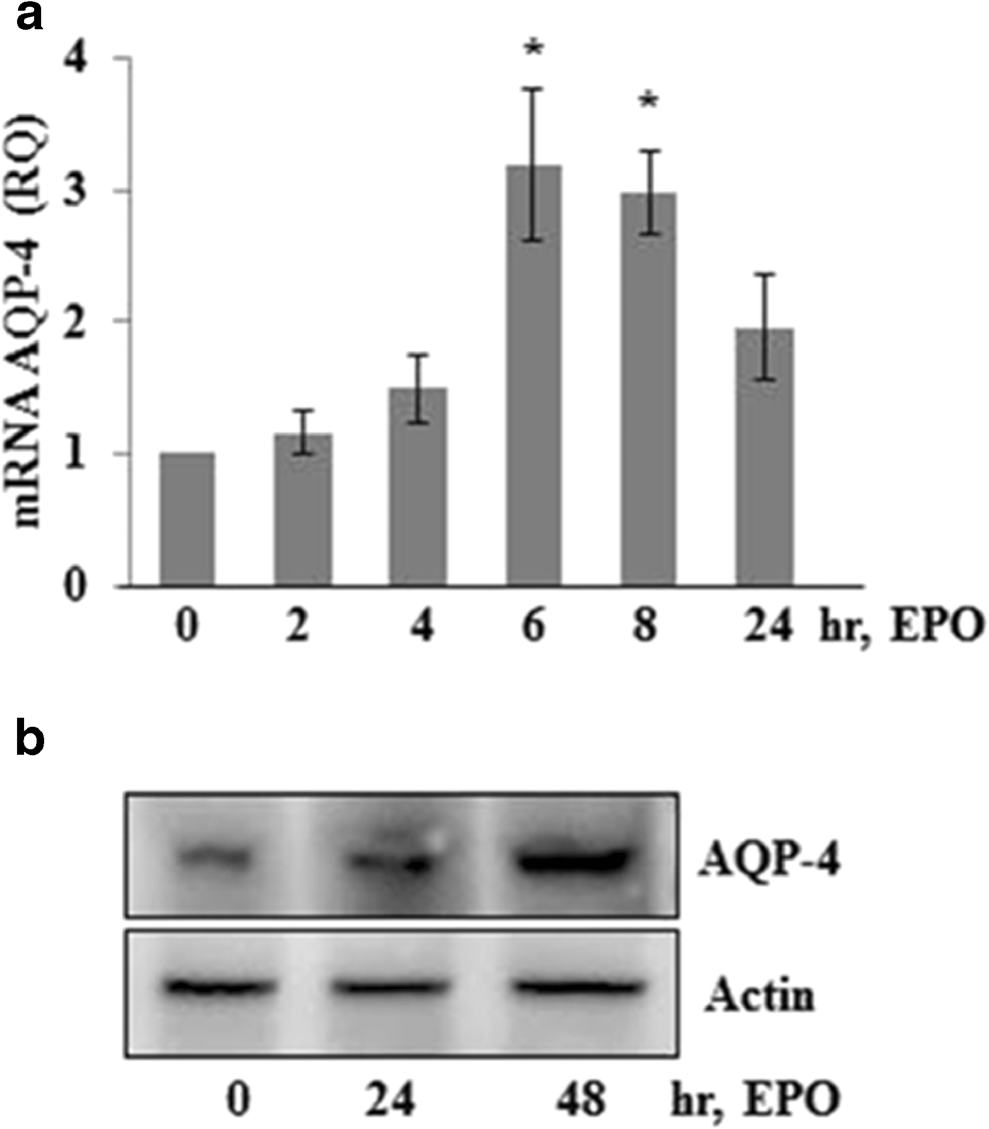
AQP4 expression in cultured astrocytes after EPO treatment. (A) Astrocytes were treated with EPO for indicated time points and AQP4 expression was analyzed with RT-PCR whereas by Western blot in (B). The same blot was stripped and reprobed with anti-actin antibody for endogenous control

Since AQP4 channels are highly expressed at the end-feet of astrocytes and play indispensable role in clearing water via trans-parenchymal pathway into the cerebral vascular system, we investigated the level of AQP4 in cultured astrocytes in response to EPO. Our data show that EPO challenge increases AQP4 expression in astrocytes (Fig. 3(A), B).

### Effect of EPO on AQP4 targeting miRNAs

**Fig. 4:**
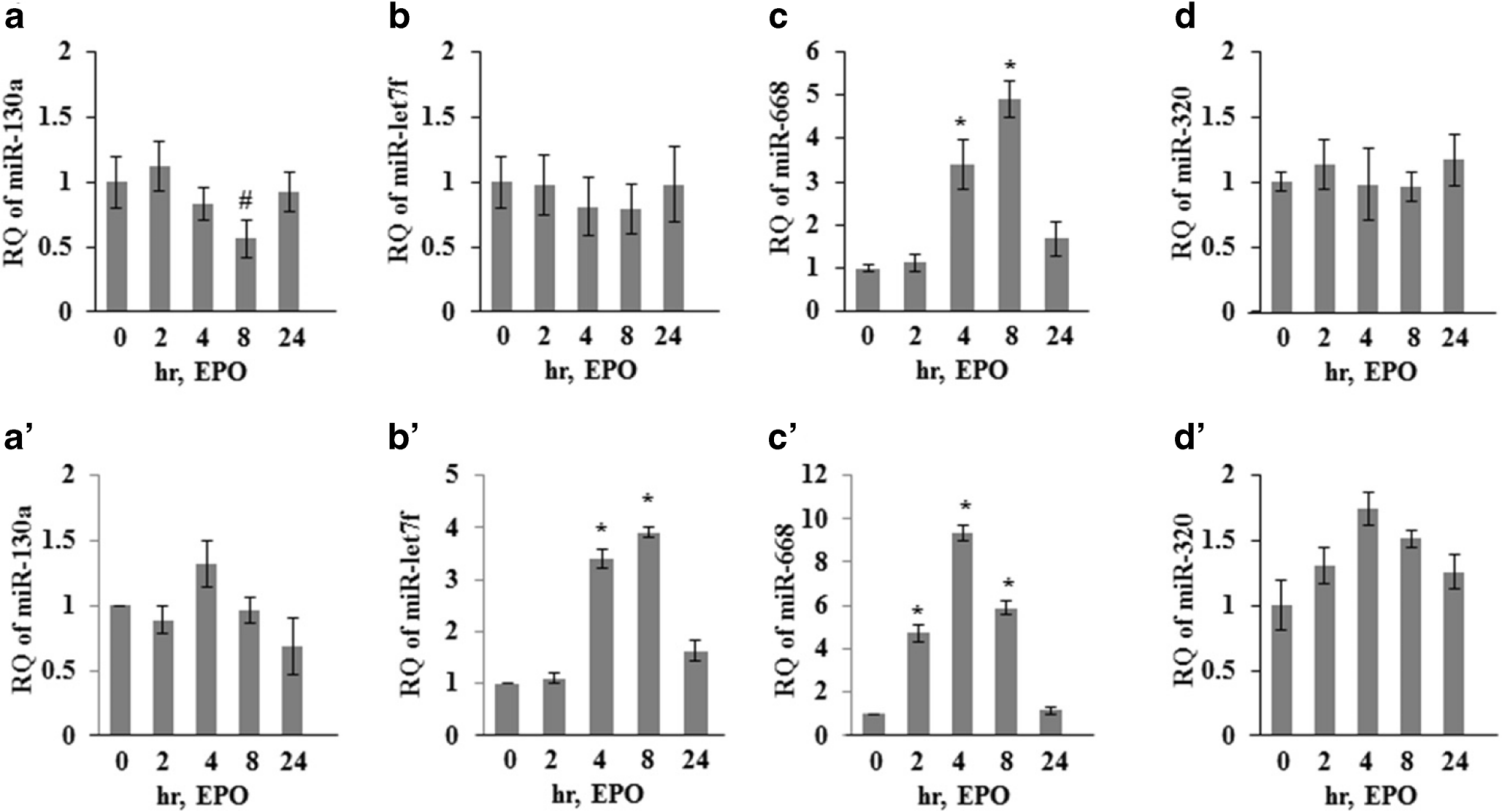
EPO positively regulates AQP4 targeting miR-668 in CP ependymal cells and in astrocytes: quantitative RT-PCR showing miR-130a, miR-let-7f, miR-668, and miR-320 in ependymal cells derived from CP (A, B, C, and D) and in astrocytes (A’, B’, C’, and D’) treated with of 20 IU/ml of EPO for 0, 2, 4, 8, and 24 h. Total RNA was isolated and expression of different miRNAs was determined by qPCR. The data shown here are from a representative of three experiments ±SEM. *p < 0.01, **#***p* < 0.05 vs untreated group

**Fig. 5:**
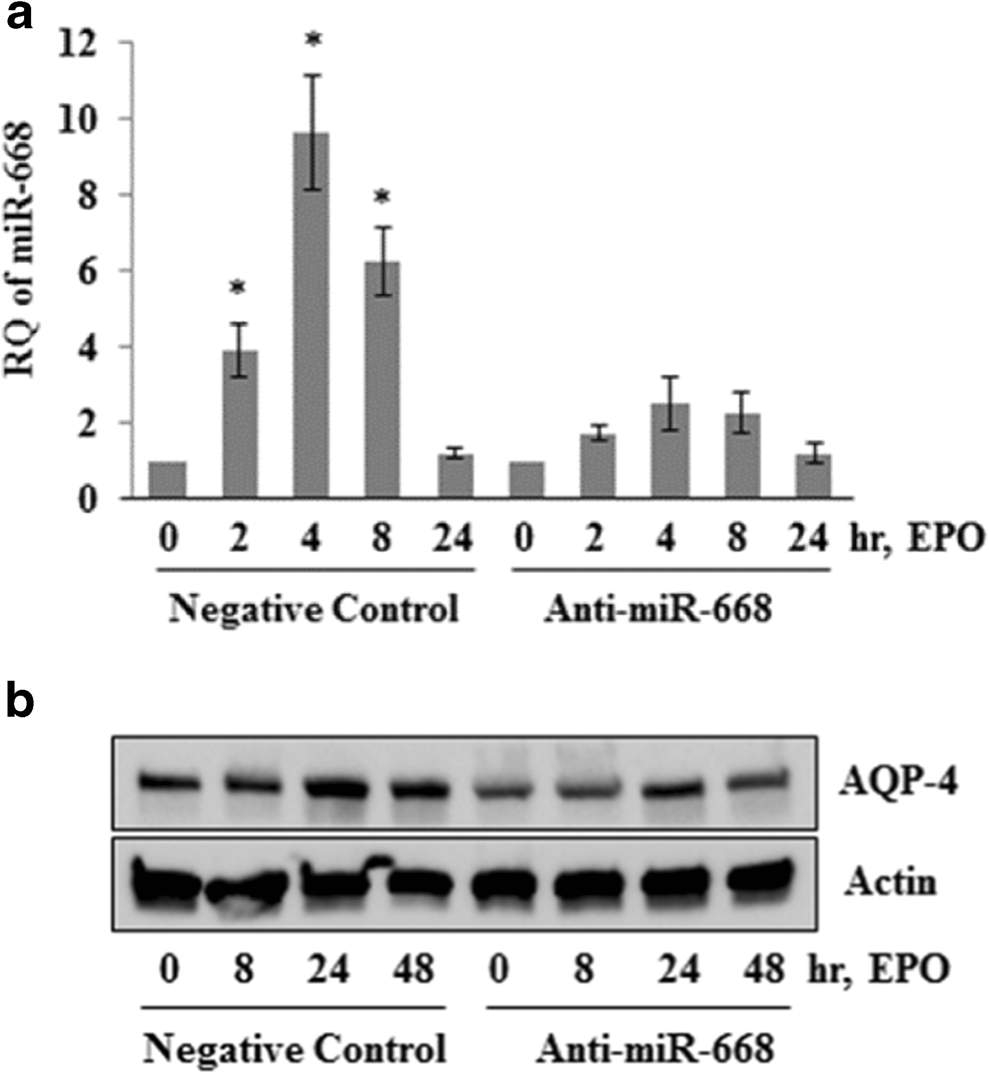
Anti-miR-668 dampened EPO effect on AQP4 expression. (A) Astrocytes were transfected with antimiR-668 or anti-miRNA negative control. After 48 h of transfection, cells were treated with EPO (20 IU/ml) for 0, 2, 4, 8, and 24 h. Total RNA was extracted, and the miR-668 levels were determined using RT-PCR. The relative expression levels of miR-668 were determined with respect to the negative controls. The data shown here are from a representative of three experiments ± SEM. *p < 0.01, **#***p* < 0.05 vs untreated group. (B) The corresponding AQP4 levels were also determined. Astrocytes were transfected with anti-miR-668 or anti-miRNA negative control. After 48 h of transfection, cells were treated with EPO (20 IU/ml) for 0, 24, and 48 h. Total protein was extracted and subjected to Western blotting to determine the AQP4 protein levels. The same blot was reprobed with anti-actin antibody for endogenous control. The data shown here are from a representative of three experiments

In Z310 cells, EPO significantly decreased miR-130a and increased miR-668 expression while there was no change in miR-320a and miR-let7f levels (Fig. 4(A–D)). In astrocyte, the levels of miR-668 and miR-let7f significantly upregulated with EPO treatment while no substantial change in miR-130a and miR-320a was observed (Fig. 4(A’–D’)). To establish if increased level of miR-668 in response to EPO affects the downstream target AQP4 expression, we transfected the astrocyte cells with anti-miR-668 prior to EPO treatment. As shown in Fig. 5(A, B), anti-miR-668 transfection dampened EPO-mediated upregulation of AQP4 in astrocytes. These finding supports the hypothesis that increased levels of miR-668 in response to EPO affect expression of their downstream target AQP4.

### Effects of EPO on cultured cerebrovascular endothelial cells: AQP4 expression and angiogenesis

**Fig. 6:**
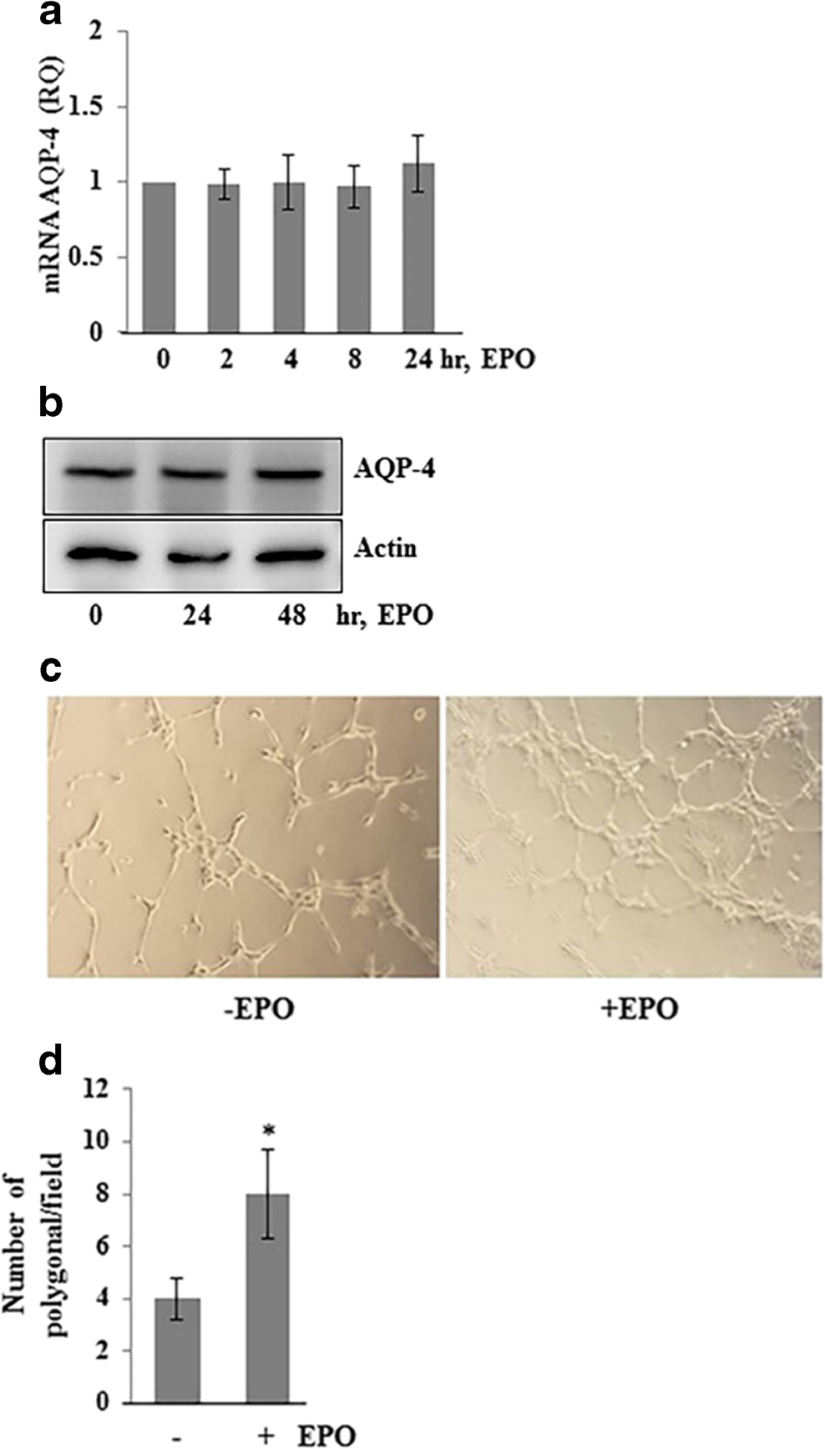
EPO-enhanced endothelial Matrigel tube formation network. (A) Confluent monolayer of HBME cells was treated with EPO (20 IU/ml) for 0, 2, 4, 8, and 24 h. Total RNA was isolated and AQP4 expression was analyzed with RT-PCR. (B) Confluent monolayer of HBME cells was treated with EPO (20 IU/ml) for 0, 24, and 48 h. Total protein was extracted and subjected to Western blotting to determine the AQP4 protein levels. The same blot was reprobed with anti-actin antibody for endogenous control. The blots are representative of three different experiments. (C) HBME cells were seeded on growth factor reduced Matrigel and treated without and with EPO (20 IU/ml) in serum-free, phenol red-free media. Representative images (× 20) show the network formed by HBME cells. (D) Polygonal area of network was counted in each field. The data shown here are from a representative of three experiments ± SEM. *p < 0.05

EPO treatment had no effect on AQP4 level in HBME cells (Fig. 6(A, B)), however significantly increased tube network formation of ECs as compare to untreated ECs (Fig. 6(C, D)). Thus, we have identified a novel role of EPO in enhancing AQP4 expression in astrocytes and ependymal cells, and triggering endothelial angiogenesis, which plays a key role in regulating CSF equilibrium.

## Discussion

Present study examines the role for EPO in modulating AQP4 water channels in experimental hydrocephalus. It is noted that EPO treatment upregulates AQP4 expression and reduces dilated cerebral ventricles in kaolin-induced model of obstructive hydrocephalus in rat pups. Also, we identified the role of miR-668 as an endogenous modulator of AQP4 expression in response to EPO.

Earlier studies by Tourdias et al. showed an upregulation of AQP4 in astrocytes in response to communicating hydrocephalus, which may participate in clearance of excess fluid to the blood stream [12]. On the other hand, Mao et al., who studied effects of obstructive hydrocephalus on AQP4 expression in rat, found changes in mRNA level but not in protein level of AQP4 channel [4]. In our study, we did not observe substantial difference in AQP4 expression between normal and obstructive hydrocephalic brain. This observation suggests that differences in AQP4 expression might depend on the model and severity of hydrocephalus.

Malformation of ependymal epithelial cells has been implicated in the etiology of hydrocephalus [1, 25]. Consistent with these observations, we found denudation of ependymal structure after kaolin injection, which dramatically reduced after EPO treatment likely due to decreased intracranial pressure (ICP) and ventriculomegaly. Thus, the ependymal layer denudation is the result of pressure hydrocephalus and appears to be reversible, parallel to the reduction in ventricular size following the EPO treatment. We found an increased expression of AQP4 in the ependymal layer in vivo as well as cultured astrocytes following EPO treatment. Therefore, upregulation of AQP4 expression in the ventricle ependymal layer could contribute to clearing of CSF out of the ventricle into parenchyma. Since AQP4 proteins are also located at astrocyte end-feet, we hypothesized that their upregulation could facilitate the removal of excessive water from the parenchyma into the blood thereby promoting EPO-mediated CSF absorption. This in turn would reduce hydrocephalus by upregulating AQP4 expression at major junctions of fluid compartments. Our findings are in line with previous observation showing that EPO-mediated increase in expression around hematoma helps in reducing brain swelling [16].

In this study, we demonstrate that EPO decreases miR-130a and increases miR-668 which in turn upregulates AQP4 expression in cultured ependymal epithelial cells and astrocytes. Inhibition of miR-668 by anti-miR-668 prevents the EPO-mediated upregulation of AQP4. AQP4 has been shown to be a direct target of miR-320a and miR-130a and under ischemic condition, delivery of anti-miR-320a and anti-miR-130a upregulates AQP4 expression and exhibits beneficiary effect on infarct size [18, 19]. However, we observed miR-668 as a strong activator of AQP4 expression in response to EPO. This positive regulation of AQP4 expression by miR-668 may be oversimplistic, since other signaling molecules down to erythropoietin receptor (EPOR) may be involved in AQP4 expression [26, 27]. Further investigation is needed of the roles of microRNA in hydrocephalus physiology and management.

Endothelium transports water from peri-vascular astrocyte end-feet into vessel lumen by diffusion and vesicles mediated transport. Pro-angiogenetic property of EPO has been well documented [28]. In our experiment, when cultured HBME cells treated with EPO in in vitro, we did not observe any changes in AQP4 expression in endothelial cells but noted increased endothelial tube formation. Therefore, we hypothesized that increased number of vessels may potentiate the exit of water from peri-vascular astrocyte end-feet into blood vessel.

## Conclusion

Our experiments show that (i) EPO-mediated upregulation of AQP4 significantly reduces dilation of the cerebral ventricles in obstructive hydrocephalus pups; (ii) EPO triggers AQP4 expression in ependymal cells and astrocytes; (iii) endothelial angiogenesis induced by EPO is likely the mechanism of enhanced CSF resorption into the blood vessel; and (iv) EPO may represent a feasible approach to potential therapeutic options for hydrocephalus.
